# The Virioneuston: A Review on Viral–Bacterial Associations at Air–Water Interfaces

**DOI:** 10.3390/v11020191

**Published:** 2019-02-22

**Authors:** Janina Rahlff

**Affiliations:** University of Duisburg-Essen, Biofilm Centre, Universitätsstraße 5, 45141 Essen, Germany; Janina.rahlff@uol.de; Tel.: +49-0201-183-7081

**Keywords:** surface microlayer, air–sea interaction, surfactants, aerosols, bubbles, particles, phages, bacterioneuston, transparent exopolymer particles (TEP)

## Abstract

Vast biofilm-like habitats at air–water interfaces of marine and freshwater ecosystems harbor surface-dwelling microorganisms, which are commonly referred to as neuston. Viruses in the microlayer, i.e., the virioneuston, remain the most enigmatic biological entities in boundary surface layers due to their potential ecological impact on the microbial loop and major air–water exchange processes. To provide a broad picture of the viral–bacterial dynamics in surface microlayers, this review compiles insights on the challenges that viruses likely encounter at air–water interfaces. By considering viral abundance and morphology in surface microlayers, as well as dispersal and infection mechanisms as inferred from the relevant literature, this work highlights why studying the virioneuston in addition to the bacterioneuston is a worthwhile task. In this regard, major knowledge gaps and possible future research directions are discussed.

## 1. Introduction

Due to its exclusive position between the atmosphere and the hydrosphere and by spanning about 70% of the Earth’s surface, the sea-surface microlayer (sea-SML) is regarded as a fundamental component in air–sea exchange processes and in biogeochemical cycling [1]. Although having a minor thickness of <1000 µm [2], the elusive SML is long known for its distinct physicochemical characteristics compared to the underlying water [3], e.g., by featuring the accumulation of dissolved and particulate organic matter [3,4], transparent exopolymer particles (TEP), and surface-active molecules [5,6]. Therefore, the SML is a gelatinous biofilm [7], maintaining physical stability through surface tension forces [8]. It also forms a vast habitat for different organisms, collectively termed as neuston [9] with a recent global estimate of 2 × 10^23^ microbial cells for the sea-SML [10].

Life at air–water interfaces has never been considered easy, mainly because of the harsh environmental conditions that influence the SML [11]. However, high abundances of microorganisms, especially of bacteria and picophytoplankton, accumulating in the SML compared to the underlying water were frequently reported [4,12,13], accompanied by a predominant heterotrophic activity [14,15,16]. This is because primary production at the immediate air–water interface is often hindered by photoinhibition [17,18]. However, some exceptions of photosynthetic organisms, e.g., *Trichodesmium, Synechococcus*, or *Sargassum,* show more tolerance towards high light intensities and, hence, can become enriched in the SML [4,19,20]. Previous research has provided evidence that neustonic organisms can cope with wind and wave energy [12,21,22], solar and ultraviolet (UV) radiation [23,24,25], fluctuations in temperature and salinity [26,27], and a higher potential predation risk by the zooneuston [28]. Furthermore, wind action promoting sea spray formation and bubbles rising from deeper water and bursting at the surface release SML-associated microbes into the atmosphere [29]. In addition to being more concentrated compared to planktonic counterparts, the bacterioneuston, algae, and protists display distinctive community compositions compared to the underlying water, in both marine [8,19,20,21,30,31] and freshwater [32,33] habitats. Furthermore, the bacterial community composition was often dependent on the SML sampling device being used [34,35,36]. While being well defined with respect to bacterial community composition, little is known about viruses in the SML, i.e., the virioneuston. This review has its focus on virus–bacterium dynamics at air–water interfaces, even if viruses likely interact with other SML microbes, including archaea and the phytoneuston, as can be deduced from viral interference with their planktonic counterparts (reviewed by Brussaard [37] and Rohwer and Thurber [38]). Although viruses were briefly mentioned as pivotal SML components in a recent review on this unique habitat [39], a synopsis of the emerging knowledge and the major research gaps regarding bacteriophages at air–water interfaces is still missing in the literature.

## 2. Viruses as Overlooked Key Players in the SML Realm

Viruses are the most abundant biological entities in the water column of the world’s oceans [40]. Moreover, they are known as integral parts of global biogeochemical cycles [41], to shape and drive microbial diversity [42] and to structure trophic networks [43]. Like other neuston members, the virioneuston likely originates from the bulk seawater. For instance, Baylor et al. [44] postulated adsorption of viruses onto air bubbles as they rise to the surface [44], or viruses can stick to organic particles [45] also being transported to the SML via bubble scavenging [46].

**Figure 1:**
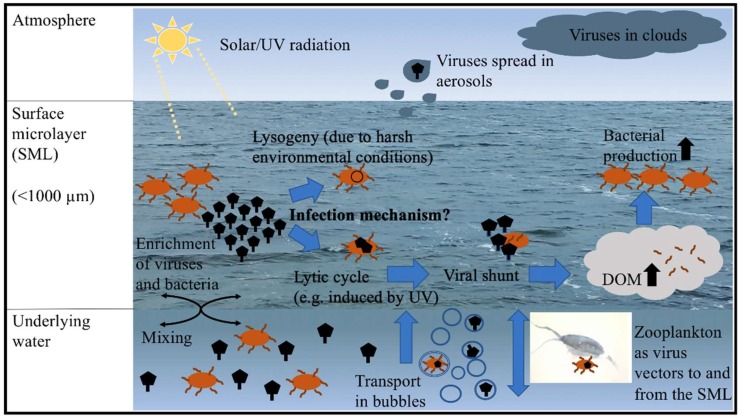
Viral–bacterial dynamics in the surface microlayer (SML) and beyond. DOM = dissolved organic matter, UV = ultraviolet.

Within the SML, viruses interacting with the bacterioneuston will probably induce the viral shunt, a phenomenon that is well known for marine pelagic systems. The term viral shunt describes the release of organic carbon and other nutritious compounds from the virus-mediated lysis of host cells, and its addition to the local dissolved organic matter (DOM) pool [47]. The enriched and densely packed bacterioneuston forms an excellent target for viruses compared to the bacterioplankton populating the subsurface. This is because high host-cell numbers will increase the probability of host–virus encounters. The viral shunt might effectively contribute to the SML’s already high DOM content enhancing bacterial production as previously suggested for pelagic ecosystems [43] and in turn replenishing host cells for viral infections (Figure 1). By affecting the DOM pool, viruses in the SML might directly interfere with the microbial loop being initiated when DOM is microbially recycled, converted into biomass, and passed along the food web. In addition, the release of DOM from lysed host cells by viruses contributes to organic particle generation [48]. However, the role of the virioneuston for the microbial loop has never been investigated.

Not only DOM but also organic particles show frequent enrichments within the SML [49], and Bigg et al. [50] reported that SML particles from the central Arctic Ocean were loaded with viruses. Van der Waals forces are responsible for the adsorption of phages to colloid materials [51], and Weinbauer et al. [52] suggested that aggregates could be net scavengers of attached viruses reducing the abundance of free-living ones [53]. Viruses were also found attached to TEPs along trophic gradients in the southwestern lagoon of New Caledonia [45]. The authors showed a positive correlation of TEP size and viral abundance, and further proposed TEPs being hot spots for viral infections [37]. Considering that unballasted TEPs can be highly enriched within the SML due to their lower density than seawater [13,54,55], studying the interactions between SML and TEPs and the virioneuston becomes highly relevant. Organic-particle aggregation and vertical transport may have crucial implications for carbon cycling and viral dispersal either by buoyant TEPs accumulating in the SML or by sinking TEPs falling to the deep sea.

Enrichment of surfactants in the SML was observed even at considerable high oceanic wind speeds up to ~13 m s^−1^ [56]. Surfactants can be either produced by phytoplankton [40] or marine bacteria [57]. Typical operational taxonomic units sequenced from the SML were assigned to *Bacillus* spp. or *Acinetobacter* spp. and were strongly associated with surfactants [58]. Interestingly, some surface-active agents produced by marine bacteria, such as pumilacidin, which is derived from *Bacillus pumilus*, possess antiviral properties [59]. The surfactant surfactin produced by *Bacillus subtilis* showed in vitro inactivation of a broad range of viruses and worked more efficiently against enveloped than non-enveloped ones [60]. The underlying mechanism includes the disruption of the viral lipid membrane and partially the capsid [60]. Since some water- and airborne viruses from the SML bear envelopes [61], they might become targets of surfactants. However, a deeper knowledge about virus–host–surfactant interactions is required to reveal if the SML could be a source for new powerful antiviral chemicals.

In sufficient quantities, surfactants can suppress air–sea gas exchange across the SML [62,63]. Consequently, enhanced viral lysis of bacterial and algal host cells would result in the release of more surfactants and nutrients from dead cells, and might be particularly profound for virus-mediated termination of large surface blooms [64]. The increase in organics enhances turnover and production of new bacteria including those able to actively produce surfactants. These processes could theoretically reinforce the gas-exchange suppressive effect exerted by surfactants and reduce gas transfer across the SML. Doubtlessly, this will be of particular importance for climate-relevant gases such as carbon dioxide, whose transfer across the air–sea boundary has a known dependency on biogenic surfactants [65]. The role of viruses on air–sea gas exchange by the induction of bacterial and algal surfactant release, due to increased mortality but also as a potential antiviral defense mechanism of living cells, has found no attention to date.

Furthermore, bacteria are often found in high abundances within slicks, which are visual surface films forming at calm sea states when high surfactant loads dampen the energy of small waves [66]. Wurl, Stolle, Van Thuoc, Thu, and Mari [13] found that slicks have a high microbial biomass and the bacterial community composition was different from the underlying water when compared to non-slick areas. Whether slicks also differ from non-slicky SML regarding their viral communities remains to be determined. At least, certain types of slicks were formerly linked to cyanobacterial blooms such as of *Trichodesmium* sp. [67,68] or *Synechococcus* sp. [14]. The presence of cyanobacterial slicks at the air–sea boundary might be in accordance with a recent mesocosm study where different *Synechococcus* and *Prochlorococcus* phages were observed in aerosols, SML, and bulk water [61].

Microorganisms additionally become enriched in patchy surface foams [69], essentially originating from wind-induced compression of the SML [70], as well as from rising bubbles that accumulate at the surface but do not burst instantaneously [71]. Whether viruses benefit from the increased microbial biomass enriched in slicks and foams by following the “kill-the-winner” theorem [72], i.e., infecting the most abundant prokaryotes and thus maintaining bacterial diversity, remains another yet-to-be-answered question. Effects on the prokaryotic community composition should be considered in this context.

## 3. Viral Abundance and Morphotypes in the SML

In the free water column, the virioplankton typically outnumbers the bacterioplankton by one order of magnitude reaching typical bulk water concentrations of 10^7^ viruses mL^−1^ [41]. Research on the virioneuston is still in its infancy. The current body of literature shows that viruses, like their hosts, are sometimes enriched in the SML over underlying water (Table 1), though results are overall inconclusive. This may be because sampled bulk water depths varied considerably between studies (0.15–20 m), and sampling of viruses from the neuston has been conducted with a range of sampling devices (Table 1). The glass-plate sampler and the mesh screen were both regarded as suitable tools for virus sampling [35], but more comparative studies for the various types of sampling methods and with a focus on virus sampling would be desirable.

A study by Tapper and Hicks [73] found a viral abundance range of 0.1–9 × 10^6^ viruses mL^−1^ for Lake Superior (North America) with 2–15 times more viruses occurring in the upper 20 µm layer compared to water from 20 m beneath the surface. Joux et al. [74] studied the enrichment of virus-like particles (VLPs) at two coastal sites in the northwestern Mediterranean Sea and found contrasting results for enrichment factors (EF)—defined as the ratio of a species’ concentration in the SML relative to its concentration in the underlying water [75]. The EF of VLPs was close to 1 (no enrichment) at both sites during three sampling time points except for one time point, where an EF of 5.1 was recorded. Absolute counts of VLPs were in a range of 0.3–204 × 10^6^ viruses mL^−1^ as determined by VLP enumeration under the microscope. Likewise, Kuznetsova et al. [76] reported slight enrichments of VLPs for the SML (EF on average ~1.5), and VLP concentrations in the range of 1–10 × 10^6^ viruses mL^−1^ along a transect from coastal Massachusetts to the open ocean waters of the Sargasso Sea (Table 1). A highly significant, positive correlation between viral abundance in the SML and the underlying water from the various studies shown in Table 1 (Spearman rank correlation coefficient *r_s_* = 0.825, two-tailed analysis: *p* < 0.0001, *n* = 44) possibly supports the previous assumption of SML viruses originating from the underlying bulk water.

However, correlations between viral and host abundance in the SML were not apparent [74,76] as formerly shown for marine pelagic systems [77]. Probably environmental factors and/or the bacterial community composition drive the prevailing virus–host dynamics. The virus to bacteria ratio was previously shown to be ~10-fold higher in freshwater than in marine environments [78], but due to the limited amount of data, more work is certainly needed to confirm these trends for the SML. According to field data from previous studies (Table 1), virus to bacteria ratios ranged from 0.1 to 139 (median = 1.5) and from 0.03 to 29 (median = 1.8) in the SML and underlying water, respectively. Data from the mesocosm study [61] were excluded from this calculation as SML bacterial cell counts often reach unusually large numbers in artificial experimental setups [79] and, hence, might not be representative. The median values illustrate that viruses were only slightly more abundant than bacteria in both SML and subsurface if primarily marine samples were considered. A speculative reason for this observation could be that most viruses, despite relatively high bacterial numbers, reside in a non-infectious state within host cells and hence follow a strategy known as “piggyback the winner” [80] contrasting the “kill-the-winner” theorem [72]. Likewise, the harsh environmental conditions at the air–sea boundary could effectively prevent a switch to the lytic cycle. However, according to the calculated ratio, the same theory would then apply for the slightly deeper surface water. Another explanation could be that only certain viruses can tolerate SML conditions, and that they have a very narrow host range. An infection of only certain, perhaps, low-abundant host strains would also prevent viruses from reaching high numbers. Thus, to fully understand the underlying viral–bacterial dynamics, the virioneuston should best be analyzed together with the host-community composition.

The usual size range of marine viral particles ranges between 20 and 200 nm [41]. In one study, smaller particles between 20 and 60 nm were reported for virioneuston members of the central Arctic Ocean in summer 2001 [50]. Studies on the role of environmental factors such as high solar irradiance on VLP size in the SML would be useful but do not exist so far. Tapper and Hicks [73] found that about 70% of the total free viruses in the SML and subsurface water from Lake Superior were tailed by using transmission electron microscopy (TEM). The typical (polyhedral) head and tail morphology of the free viruses led the authors to assume that the viruses were bacteriophages. Head sizes were in the range of 10–70 nm, tails ranged from 10 to 110 nm. Most free viruses had a head size <60 nm and a tail. Based on TEM observations on SML samples, Ram et al. [81] found that the SML of a tropical coastal ecosystem was dominated by myoviruses (42%), which include bacteriophages possessing a contractile tail and broad host ranges [82]. Also, metagenomic sequencing revealed tailed bacteriophages of the Caudovirales (including Myoviridae and Podoviridae) in the SML in a mesocosm study [61]. Whether the viral morphologies fulfill an adaptive function for the virioneuston to inhabit in the SML or benefit interference with a particular host type requires more research on the structural features of different virioneuston members.

**Table 1:** Bacterial and viral abundances and ratios in the surface microlayer (SML) and underlying water (ULW). If *n* > 1, either ranges of minimum to maximum values are given in parentheses or ± standard deviation obtained from the reference source. EF = enrichment factor.

Viral Abundance (×10^6^ mL^−1^)		EF	*n*	Bacterial Abundance (×10^6^ cells mL^−1^)		EF	*n*	Virus to Bacteria Ratio		Sampling Site	SML Sampling Device	ULW Depth (m)	Reference
SML	ULW	SML/ULW		SML	ULW	SML/ULW		SML	ULW				
13.2 ± 1.9	16.8 ± 3.2	0.8	3	8.6 ± 0.018	7.3 ± 0.10	1.2	3	1.5	2.3	Mesocosm experiment, day 7 (Pacific seawater)	Glass plate	0.9	[61]
12.1 ± 2.2	6.0 ± 1.0	2.0	3	9.6 ± 0.015	9.9 ± 0.06	1.0	3	1.3	0.6	Mesocosm experiment, day 8 (Pacific seawater)	Glass plate	0.9	[61]
12.1 ± 2.2	19.3 ± 2.1	0.6	3	9.8 ± 0.081	8.8 ± 0.12	1.1	3	1.2	2.2	Mesocosm experiment, day 9 (Pacific seawater)	Glass plate	0.9	[61]
14.3 ± 2.8	11.0 ± 3.8	1.3	3	11.7 ± 0.11	10.0 ± 0.051	1.2	3	1.2	1.1	Mesocosm experiment, day 10 (Pacific seawater)	Glass plate	0.9	[61]
20.8 ± 2.7	29.9 ± 3.6	0.7	3	7.2 ± 0.015	10.0 ± 0.015	0.7	3	2.1	3.0	Mesocosm experiment, day 16 (Pacific seawater)	Glass plate	0.9	[61]
35.4 ± 4.2	23.6 ± 2.6	1.5	3	10.9 ± 0.17	9.8 ± 0.31	1.1	3	3.2	2.4	Mesocosm experiment, day 17 (Pacific seawater)	Glass plate	0.9	[61]
38.5 ± 5.1	29.1 ± 4.2	1.3	3	12.0 ± 0.35	11.0 ± 0.24	1.1	3	3.2	2.6	Mesocosm experiment, day 18 (Pacific seawater)	Glass plate	0.9	[61]
39.0 ± 4.2	43.6 ± 3.6	0.9	3	8.6 ± 0.071	8.4 ± 0.12	1.0	3	4.5	5.2	Mesocosm experiment, day 21 (Pacific seawater)	Glass plate	0.9	[61]
30.4 ± 4.3	79.7 ± 7.9	0.4	3	14.7 ± 0.091	12.4 ± 0.44	1.2	3	2.1	6.4	Mesocosm experiment, day 22 (Pacific seawater)	Glass plate	0.9	[61]
36.6 ± 3.5	47.6 ± 6.5	0.8	3	33.5 ± 0.26	18.6 ± 0.75	1.8	3	1.1	2.6	Mesocosm experiment, day 23 (Pacific seawater)	Glass plate	0.9	[61]
39.6 ± 5.3	36.3 ± 5.2	1.1	3	33.3 ± 0.039	29.8 ± 1.27	1.1	3	1.2	1.2	Mesocosm experiment, day 24 (Pacific seawater)	Glass plate	0.9	[61]
7.3 (4.1–18.4)	3.1 (2.0–4.9)	2.4	16	1.3 (0.5–2.9)	0.6 (0.5–1.0)	2.2	16	6.4	5.0	Halong Bay, Vietnam, Oct. 2012	Glass plate	1.5	[81]
125 (46–204)	25 (24–26)	5.1	2	0.9 (0.9–0.9)	0.9 (1.0–0.9)	1.0	2	138.9	27.8	Mediterranean Sea, Barcelona Site, Sept. 2001	Metal screen	0.5	[74]
1.8 (1.0–2.5)	1.6 (1.1–2.1)	1.1	2	2.6 (1.7–4.0)	2.4 (1.3–3.9)	1.2	2	0.7	0.7	Mediterranean Sea, Barcelona Site, Mar. 2002	Metal screen	0.5	[74]
24.5	25.8	0.9	1	1.0 (0.9–1.1)	0.9 (0.9–1.0)	1.1	2	24.5	28.7	Mediterranean Sea, Banyuls Site, Sept. 2001	Metal screen	0.5	[74]
0.4 (0.3–0.4)	0.3 (0.3–0.4)	1.1	3	0.9 (0.8–1.2)	1.0 (0.8–1.1)	0.9	3	0.4	0.3	Mediterranean Sea, Banyuls Site, Mar. 2002	Metal screen	0.5	[74]
10.9	1.4	7.8	1	12.4	6.2	2.0	1	0.9	0.2	Stony Brook Harbor, NY, June 2003	Polyester screen	0.15	[83]
2.4	3.1	0.8	1	2.0	1.7	1.2	1	1.2	1.8	Stony Brook Harbor, NY, July 2003	Polyester screen	0.15	[83]
2.4	0.9	2.7	1	2.7	1.6	1.7	1	0.9	0.6	North Atlantic Ocean, Sample No. 1, June 2001	Polyester screen	0.15	[76]
1.9	1.7	1.1	1	1.9	1.5	1.2	1	1.3	1.1	North Atlantic Ocean, Sample No. 2, June 2001	Polyester screen	0.15	[76]
2.8	3.9	0.7	1	1.6	1.5	1.1	1	1.5	2.7	North Atlantic Ocean, Sample No. 3, June 2001	Polyester screen	0.15	[76]
5.5	2.9	1.9	1	2.0	1.8	1.1	1	1.2	1.6	North Atlantic Ocean, Sample No. 4, June 2001	Rotating drum	0.15	[76]
1.6	1.0	1.6	1	1.0	1.1	0.9	1	2.4	0.9	North Atlantic Ocean, Sample No. 5, June 2001	Polyester screen	0.15	[76]
4.6	2.2	2.0	1	1.3	0.7	1.7	1	1.9	3.1	North Atlantic Ocean, Sample No. 6, June 2001	Polyester screen	0.15	[76]
3.0	1.2	2.4	1	0.7	0.6	1.1	1	3.6	2.0	North Atlantic Ocean, Sample No. 7, June 2001	Polyester screen	0.15	[76]
2.4	2.0	1.2	1	0.7	0.6	1.2	1	3.5	3.4	North Atlantic Ocean, Sample No. 8, June 2001	Polyester screen	0.15	[76]
2.5	2.4	1.0	1	1.2	0.8	1.5	1	2.1	3.1	North Atlantic Ocean, Sample No. 9, June 2001	Polyester screen	0.15	[76]
3.2	2.8	1.2	1	0.9	1.0	0.9	1	2.7	2.7	North Atlantic Ocean, Sample No. 10, June 2001	Rotating drum	0.15	[76]
3.1	4.1	0.8	1	1.3	0.9	1.4	1	1.9	4.5	North Atlantic Ocean, Sample No. 11, June 2001	Polyester screen	0.15	[76]
3.8	3.9	1.0	1	1.0	0.8	1.2	1	2.5	4.9	North Atlantic Ocean, Sample No. 12, June 2001	Polyester screen	0.15	[76]
2.3	3.5	0.7	1	0.7	0.8	0.9	1	3.4	4.5	North Atlantic Ocean, Sample No. 14, June 2001	Rotating drum	0.15	[76]
6.9	3.8	1.8	1	1.3	0.9	1.5	1	1.9	4.5	North Atlantic Ocean, Sample No. 15, June 2001	Polyester screen	0.15	[76]
3.8	3.4	1.1	1	2.2	0.9	2.3	1	1.1	3.7	North Atlantic Ocean, Sample No. 16, June 2001	Polyester screen	0.15	[76]
1.9	1.7	1.1	1	1.3	1.2	1.1	1	1.9	1.4	North Atlantic Ocean, Sample No. 17, June 2001	Rotating drum	0.15	[76]
2.8	1.7	1.7	1	1.9	1.3	1.4	1	1.3	1.3	North Atlantic Ocean, Sample No. 18, June 2001	Polyester screen	0.15	[76]
3.5	3.2	1.1	1	1.2	1.0	1.2	1	2.0	3.3	North Atlantic Ocean, Sample No. 19, June 2001	Polyester screen	0.15	[76]
2.7	2.0	1.4	1	1.5	1.4	1.1	1	1.6	1.5	North Atlantic Ocean, Sample No. 20, June 2001	Rotating drum	0.15	[76]
10.9	5.1	2.1	1	2.4	1.7	1.4	1	1.0	3.1	North Atlantic Ocean, Sample No. 21, June 2001	Polyester screen	0.15	[76]
1.7	1.0	1.7	1	2.6	1.9	1.4	1	0.9	0.5	North Atlantic Ocean, Sample No. 22, June 2001	Rotating drum	0.15	[76]
3.1	2.0	1.5	1	2.4	1.7	1.4	1	1.0	1.2	North Atlantic Ocean, Sample No. 23, June 2001	Polyester screen	0.15	[76]
2.8	0.2	15.4	1	5.2	2.2	2.3	1	0.5	0.1	Lake Superior, June 1993	Teflon sheet	20	[73]
9.2	0.9	10.7	1	18.3	1.2	15.4	1	0.1	0.7	Lake Superior, July 1993	Teflon sheet	20	[73]
0.7	0.3	2.2	1	1.7	1.7	0.9	1	1.5	0.2	Lake Superior, Aug. 1993	Teflon sheet	20	[73]
1.7	0.2	11.0	1	9.2	4.6	2.0	1	0.3	0.03	Lake Superior, Oct. 1993	Teflon sheet	20	[73]

## 4. Viral Dispersal in and out of the SML

While it was shown that bacteria are actively transferred from bursting bubbles at the sea surface to sea-spray aerosols [84], the same is obviously true for viruses [44,83,85], which can even end up in clouds [86] (Figure 1). In one recent study, virus aerosolization was proposed to be less efficient than for bacteria [61]. This is because viral concentrations in aerosols were very similar to those of bacteria (1.19 × 10^7^ ± 0.58 × 10^7^ cells m^−3^), i.e., bacteria were preferentially aerosolized over viruses, or viruses were more prone to decay. Considering that aerosols originate from SML material, the observation of equal numbers of bacteria and viruses in aerosols also matches my previous calculation showing that virus to bacteria ratios expressed as a median over several field studies were in the same range for both SML and underlying water. However, there is also contradictory evidence claiming that more viruses than prokaryotes occur in experimentally produced aerosols [87]. Apparently, more conclusive work is needed on the topic.

As inferred from genomic comparisons, lipid-enveloped viruses such as Polydnaviridae and Alloherpesviridae got airborne suggesting that hydrophobic surfaces are crucial for this process [61]. This is in line with another report finding 140,000-fold enrichments of lipids in experimentally produced aerosols compared to seawater, which the authors attributed to the surface-active properties of lipids [87]. Thus, morphological characteristics could be equally important for governing virus aerosolization as environmental factors. Atmospheric transport of viruses might be an important dispersal mechanism for spreading viral infections over long distances over the ocean [88]. Interestingly, Reche et al. showed that viruses are associated with smaller aerosol particles compared to bacteria, which prolongs their atmospheric residence time [89] providing a sound explanation for the fact that identical viral sequences can occur in geographically distant environments [90,91]. While the existence of potentially pathogenic bacterial strains such as of *Legionella* and *Vibrio* in surface films has been reported [8,22,61], a potential threat for public health by the presence of pathogenic viruses and their dispersal from the SML still needs to be elucidated.

In addition, viruses were found attached to mesozooplankton, e.g., collected from the neuston of the Red Sea using a Manta neuston net that sampled the top 1 m of the water column [92]. Due to observed high mortality of copepods (phylum Arthropoda) in their samples, the authors were looking for VLPs on *Acartia fossae,* a blue-pigmented surface-dwelling copepod, and its prey, *Trichodesmium* cells. Analyses using TEM revealed different morphologies of VLPs on the copepods in contrast to the *Trichodesmium* cells. Metatranscriptomic analyses showed that genes responsive to virus reproduction showed increased expression in these copepods. Therefore, this study provided a first important indication for the interaction between viruses and higher, non-prokaryotic trophic levels. This aspect deserves further investigation, as many mesozooplankton species frequently populate the SML [4,93] and hence represent appropriate bacterial and viral targets. Zooplankton species that occasionally migrate to the SML during diel vertical migration or for feeding purposes [28] might also serve as important vectors for viruses that infect bacteria or phytoplankton living therein [94] (Figure 1).

## 5. Infection Mechanisms of the Virioneuston

Most marine viruses in surface water are infective [95], but contradictory assumptions exist on the prevailing viral infection mode for the SML. On the one hand, due to the stressful environmental conditions associated with populating the SML [11], lysogeny as a survival mechanism might be the preferred viral infection strategy [73]. The extent of lysogeny, i.e., the integration of the viral genome (the prophage) into the host genome, can be experimentally estimated by induction of prophages into lytic phages using UV radiation or the antibiotic mitomycin C [96]. Tapper and Hicks [73] reported on higher amounts of temperate prophages in the SML (0.1%–7.4%) compared to the underlying water (0.1%–1.8%) in freshwater.

On the other hand, the high incidence of UV radiation at the immediate air–sea interface could induce prophages to become lytic, even though natural UV light was not always efficient to induce lysogens in surface water [97]. A range of other important environmental conditions such as sudden changes in temperature or the effect of pollutants (mainly of polyaromatic hydrocarbons, PAHs) can induce lysogenic viral production as previously demonstrated for marine and estuarine environments [98]. Since the SML is a repository for PAHs and prone to temperature fluctuations [3,99], these inducing agents may have particular relevance for prophage induction in this habitat.

High host abundance and system productivity can promote the lytic lifestyle of viruses [100]. In one case when bacterial production in the SML of Halong Bay, Vietnam was higher than in the underlying water, viral lytic infections (5.3%–30.1%) dominated over lysogeny (0%–1.4%). The same study demonstrated that certain bacterial morphotypes, namely rod-shaped cells, were particularly prone to viral lysis [81]. Since certain bacteria stick to organic particles within the SML [49], virally infected bacteria attached to particles might be masked and hard to find using microscopic approaches [81], a fact that should be considered in future virioneuston studies focusing on the detection of lytic behavior.

## 6. Future Perspectives and Conclusions

Recent advances in viral metagenomics (viromics) allow for high-throughput sequencing approaches and in silico detection of novel viruses from uncultured environmental samples. New bioinformatic tools for the identification of viral genomes based on major hallmark genes [101] and k-mer frequencies [102] and for matching hosts to viruses [103] have largely facilitated this progress (recent review by Hurwitz et al. [104]). In addition, an improved understanding on the role of clustered regularly interspaced short palindromic repeats (CRISPR) as a prokaryotic viral defense mechanism allows microbiologists to link viruses to their hosts by matching viral protospacers to host CRISPR spacers [105]. Unfortunately, only one single study used metagenomic sequencing to explore viral and bacterial communities in the SML, underlying water, and aerosol samples in a mesocosm setup [61]. More work is needed to identify which major environmental factors (turbulence resulting from wind speed, solar radiation, temperature, etc.) dominantly shape the virioneuston community and whether these influences are independent from the host community. Elucidating the major viral infection strategies during different environmental conditions, from different seasons, and latitudes, remains another open task to be addressed. Viral impacts on surfactant release and surface blooms implement influences on gas exchange and biogeochemical matter cycling and require further attention. Dispersal of airborne viruses originating from the SML, and potential hazards for the public should get more into our focus.

Air–water interfaces span more than 70% of the global surface area and therein sojourning viruses likely have profound implications for biogeochemical cycling, gas exchange, food web structure, and human health. This review illustrates that much more remains to be discovered about how the virioneuston copes with the many challenges caused by its unorthodox position between the lower atmosphere and the upper hydrosphere.
